# Liberty of the Press—Club-Law

**Published:** 1830-04-01

**Authors:** 


### LIBERTY OF THE PRESS.

It is an old observation, that those demagogues who bawl out with greatest vehemence for liberty, and denounce with direst imprecations all who would impose shackles on human actions, are the most imperious tyrants the moment they become invested with power. It is, so with the heroes of the radical press. They abuse, slander, and vilify all to whom they are opposed; but the instant that any thing like a retaliation is made on themselves, they fly to the strong arm of the law—and when the courts of justice have become too familiar with their character, then the club or the pistol is raised against any individual who attempts to pay them home in their own coin. The men of the LANCET will never more prosecute for libels. They are too well known in court; and their adversaries are now sufficiently versed in the law of libel to defend and attack, without risk of legal proceedings. Thus matched, and overmatched in fair and open literary warfare, an effort has been made to gag the medical press by powder and shot! Men, whose lives are of some value to the community, as well as to their families—whose assertions have weight with the public, because they are known to have some regard to honour and truth—men, in fine, who have a character as well as a life worth preserving, are now forsooth, to be called to Chalk-farm, by every obscure dabbler in scandal, who indites a lucubration in the radical press! According to this system of club-law, no respectable editor of a journal can expect to exist for six months, even were his aim as steady and as fatal as that of a Macnamara or a Stackpole! Individuals must succumb beneath numbers, for the hydra-headed monster of the radical press could never be decapitated by a single arm. Men, whose lives are worthless to the community, and, perhaps, a burthen to themselves—who have no characters to lose—who had rather have celebrity, even in a bad cause, than no celebrity at all, would always rise, in rapid succession, were the pistol and the bludgeon to become the arbiter of the press! Fortunately, however, we have, in this country, laws to protect us from open aggression, arms given us by nature to repel brutal assaults, and a FREE PRESS to refute calumnies and aspersions on our character.

These general remarks have been suggested by a recent and wanton assault—or at least, the threat of an assault, on the person of a private physician, whose name even is not attached to any journal, and who, by reason, common sense, and law, is not responsible for writings unauthenticated by his signature, however public rumour, with her hundred *lying* tongues, may couple his name with anonymous essays. Had any one the hardihood or indelicacy to make Sir Walter Scott responsible personally for any remarks in his various publications, till he chose to avow himself as the author of his works? No! No Englishman at least. A foreigner, indeed, we believe, did once make himself ridiculous by his *assumptions*, and was laughed at for his pains. The celebrated Scotch novelist, even when interrogated by his sovereign, did not consider it necessary or imperative to avow himself the author of works which he chose to publish anonymously. The law has provided a responsible subject for every line that issues from the press, in the person of the publisher or printer, where the author is anonymous—and no man has a right to go beyond the arm of the law. What would be thought of that Attorney General who commenced a criminal information against a *supposed* author of an anonymous libel on king or minister? And what right has Mr. M'Christie or any other individual to persecute or prosecute Dr. Macleod because he has heard that he is the editor of the Medical Gazette? We say that he had no right to do so; and had Dr. Macleod accepted a challenge upon such an assumption, he would have betrayed the liberty of the subject as well as the liberty of the press. It cannot be said that we have either personal feelings or fears on this point—for the Editor never concealed his name, and never shrunk from responsibility, legal or otherwise. But we strongly protest against the impudent and presumptuous system of calling upon an individual for personal satisfaction, merely because rumour has assigned to him the authorship of this or that production. And if Dr. Macleod had put his name on the cover of the Medical Gazette, as responsible in law for what appeared in that Journal, we do not conceive that the interests of science, the freedom of the press, or personal honour call upon him to go out, pistol in hand, with every author, reporter, or case inditer, whose feelings may be hurt by criticisms in his Journal. We admit indeed that nothing but an extreme necessity should induce a journalist to give the lie direct to the assertion of any writer, however humble his station in life, or however problematical the character of his lucubrations. But the present case was very different from that of a direct imputation on the veracity of Mr. M'Christie by the Editor of the Gazette, whoever he may be. The imputation was made on the express authority (in writing) of Mr. Earle, the party principally concerned—and if Mr. M'Christie had given himself a moment's time for sensible consideration, he would have seen the propriety of applying at once, to Mr. Earle, to know whether the allegation in the journal was correct. If Mr. Earle authorised, in writing, these accusations of falsehood against Mr. M'Christie, and acknowledged that he had done so, was *he* not the proper person to be called to account? But, no. Mr. M'Christie, instead of attacking the real, the acknowledged, the publicly named impugner of his veracity, flies to wreak his vengeance on a private individual, who is *suspected* of being the Editor of the Journal through which the accusation was made!! Was there ever such a piece of injustice, such an instance of madness, in any man pretending to common sense, or the slightest perception of what is right, proper, and decent? Here there was not the least attempt made to fix the injury on its proper author, whose name was proclaimed to the world, and who was, in all honour and justice, the responsible person. No! The penalty was to be inflicted, not on the *father* of the sin, but on the unfortunate *Accoucheur* who was *suspected* of bringing it into the world!

The cause of all this unjust and disgraceful proceeding, is clear enough. We have not the smallest doubt—no man can doubt, that Mr. M'Christie was instigated by advice, which sober reflection will convince him one day, if it has not already done so, was bad and dangerous advice. Mr. Abernethy has said—and he has said some true things in his time—that "*there is such a thing as common sense.*" Common

SENSE revails in our profession, if it is to be found upon earth. Mr. Wakley and his reporter Mr. M'Christie,—and all those concerned with the LANCET, will find, ere they die, that a system of brutal violence, and reckless injustice cannot long succeed, in any range of society—and much less in a profession whose members are generally imbued with a liberal education, and whose COMMON SENSE and keen perceptions of right and wrong will not permit them to be led far astray, even by the most vivid excitement of a temporary hallucination. Every rising sun clears away some of the haze that hovers round the character of men—and, at last, they stand in their true colours and dimensions before the public eye. It is a true sign of folly to expect that the mist of delusion can last for ever. There is no instance of such an event on record!

